# Synthesis and Properties of Bioresorbable Block Copolymers of l-Lactide, Glycolide, Butyl Succinate and Butyl Citrate

**DOI:** 10.3390/polym12010214

**Published:** 2020-01-15

**Authors:** Natalia Śmigiel-Gac, Elżbieta Pamuła, Małgorzata Krok-Borkowicz, Anna Smola-Dmochowska, Piotr Dobrzyński

**Affiliations:** 1 Centre of Polymer and Carbon Materials, Polish Academy of Sciences, 41-819 Zabrze, Poland; ngac@cmpw-pan.edu.pl (N.Ś.-G.); asmola@cmpw-pan.edu.pl (A.S.-D.); 2 AGH University of Science and Technology, Faculty of Materials Science and Ceramic, 30-059 Kraków, Poland; epamula@agh.edu.pl (E.P.); krok@agh.edu.pl (M.K.-B.)

**Keywords:** biodegradable polyester, block copolymer, succinic acid, citric acid, l-lactide, ROP, polycondensation, scaffold

## Abstract

The paper presents the course of synthesis and properties of a series of block copolymers intended for biomedical applications, mainly as a material for forming scaffolds for tissue engineering. These materials were obtained in the polymerization of l-lactide and copolymerization of l-lactide with glycolide carried out using a number of macroinitiators previously obtained in the reaction of polytransesterification of succinic diester, citric triester and 1,4-butanediol. NMR, FTIR and DSC were used to characterize the materials obtained; wettability and surface free energy were assessed too. Moreover, biological tests, i.e., viability and metabolic activity of MG-63 osteoblast-like cells in contact with synthesized polymers were performed. Properties of obtained block copolymers were controlled by the composition of the polymerization mixture and by the composition of the macroinitiator. The copolymers contained active side hydroxyl groups derived from citrate units present in the polymer chain. During the polymerization of l-lactide in the presence of polyesters with butylene citrate units in the chain, obtained products of the reaction held a fraction of highly branched copolymers with ultrahigh molecular weight. The reason for this observed phenomenon was strong intermolecular transesterification directed to lactidyl side chains, formed as a result of chain growth on hydroxyl groups related to the quaternary carbons of the citrate units. Based on the physicochemical properties and results of biological tests it was found that the most promising materials for scaffolds formation were poly(l-lactide–*co*–glycolide)–*block*–poly(butylene succinate–*co*–butylene citrate)s, especially those copolymers containing more than 60 mol % of lactidyl units.

## 1. Introduction

Currently regenerative medicine and tissue engineering are presumably the most exciting scientific activities, giving hope for our aging society to extend life span and its quality. Biocompatible and biodegradable supporting structures (scaffolds) playing a role of natural extracellular matrix, in conjunction with human cells and signaling molecules are used to restore or improve tissues functions. Aliphatic polyesters such as homopolymers and copolymers of lactides, glycolide and ε-caprolactone are the most commonly used polymeric biomaterials for scaffold manufacturing, mainly due to their well-known and proven biocompatibility and defined biodegradation course. They fully meet the basic conditions for biomedical applications, but unfortunately, they have many features that limit their possible application in tissue engineering. If synthetic copolymers are used, their properties can be generally controlled already at the synthesis stage by appropriate modification of the copolymer composition and design of chain microstructure [1,2].

From the point of view of planed use of the synthesized polymers for scaffold formation, it seems logical to obtain biodegradable polyesters using compounds that occur in the metabolic processes of the cells, which rather guarantee biocompatibility of such final polymers. Such compounds include acids: citric and succinic, i.e., essential components of a series of biochemical reactions occurring in mammalian cells, e.g., in the Krebs cycle. Trifunctional citric acid can react with alcohols or polyols to form esters without any catalysts. Poly(citric acid)s (POC) obtained on this way [3] and polymers of similar type [4] are medically attractive, cell-friendly and contain functional pendant groups. However, application of POC has limitations because of low polymerization yields and low molecular weights of the final polymers. Only after additional post-polymerization processes, after cross-linking reaction, it is possible to obtain polymers with proper mechanical properties that allow their use in tissue engineering or in implant formation. During prepolymer synthesis, pendant carboxyl and hydroxyl groups of citric acid derivatives can be partially preserved to provide inherent functionality in the bulk of the material for the conjugation of bioactive molecules [5]. Nevertheless, as it has been shown, that these polymers exhibit excellent cytocompatibility and provoke only minimal inflammation in different tissues. Preliminary in vitro evaluations showed that POC supported attachment and proliferation of human aortic smooth muscle cells, endothelial cells and 3T3 fibroblasts without any surface modifications [6,7]. POC were also found to elicit strong anti-bacterial effects [8]. However, it should be taken into account that when the amount of side carboxyl or carboxylate groups is too high, POC became cytotoxic [9]. This effect also occurs in contact with other biodegradable materials—aliphatic polyester carbonates containing high amount of carboxyl side groups [10]. 

From the point of view of biomedical applications potentially very useful seem to be citric acid derivatives copolyesters, especially those having functional hydroxyl side groups. Such a polymer is for example poly[octanediol–*co*–(citric acid)–*co*–(sebacic acid)], which was obtained by polycondensation of a mixture of dicarboxylic acids: citric and sebacic with octandiol [11]. These copolymer types have shown great suitability for cell culture applications [12].

Poly(butylene succinate) (PBS) is a well-known biodegradable aliphatic polyester and it is increasingly used in forming biodegradable packaging products [13]. Aliphatic polyesters synthesized with succinic acid and diol derivatives can be used due to their good biocompatibility. However, poly(butylene succinate) presents significant limitations in tissue engineering applications related essentially to its high semi-crystallinity and hydrophobicity. Therefore, more promising results can be obtained using copolyesters containing blocks of the chains made of succinates and also from long, much more flexible sequences. To date, a number of such copolyesters have been synthesized, for example poly(butylene succinate–*co*–butylene malate)s [14], poly(butylene succinate–*co*–cyclic carbonates)s [15]. However, it is much more difficult to obtain these types copolymers containing poly(citrate) blocks. So far, by polycondensation of citric acid and hydroxyl telechelic poly(l-lactide) a microarm star copolymer containing poly(citric acid) blocks has been obtained—it was used in the formation of micelles [16].

In our research, we decided to obtain terpolymers of l-lactide or l-lactide/glycolide with butyl succinate and butyl citrate, through the synthesis of linear oligo(butyl citrates–*co*–butyl succinate) on bulk polytransesterification reaction and then using obtained compounds as macro-initiators of l-lactide polymerization or copolymerization of l-lactide with glycolide.

## 2. Materials and Methods 

### 2.1. Materials

l-Lactide, glycolide (Huizhou Foryou Medical Devices Co., Ltd., Huizhou, China), triethyl citrate and dimethyl succinate (Sigma-Aldrich, Steinheim, Germany), were used as received. Methanol and 1,4-butanediol (Aldrich, Steinheim, Germany) were used as received. Initiators and catalysts: zirconium(IV) acetylacetonate, zinc(II) acetylacetonate monohydrate (Aldrich, Steinheim, Germany) and titanium(IV) butoxide (Alfa Aesar, Haverhill, MA, USA) were used as received.

### 2.2. Macroinitiators Synthesis—Poly(Butylene Succinate–co–Butylene Citrate)

**Figure 1:**
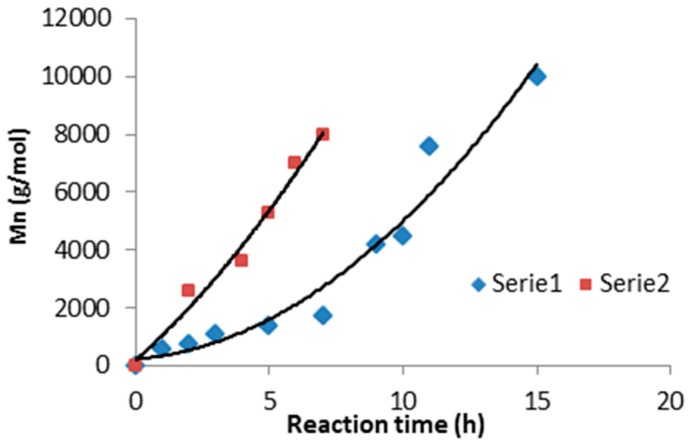
Relationship between reaction time and number average molecular weight (*M*_n_) of the synthesized polymers; Series 1—polymerization of ethyl citrate and Series 2—polymerization of methyl succinate.

The macroinitiators were obtained typically by the modified two-step polytransesterification reaction conducted in bulk [17]. The synthesis was carried out in a round-bottom flask with a volume of 0.5 dm^3^, heated by means of an oil bath, equipped with a distillation system, a thermometer, an efficient mechanical stirrer, and with connections to argon and to the vacuum pump. The first stage of the synthesis consisted of transesterification of a mixture of methyl succinate and/or triethylene citrate with 1,4-butanediol. To the dry reactor, under a cushion of argon, a measured amount of triethyl citrate, dimethyl succinate and 1,4-butanediol were loaded. In the started reaction mixture, the molar ratio of 1,4-butanediol to acid esters was as 1.07–1. Constantly stirring the reactor contents at 150 °C, the Ti(OC_4_H_9_)_4_ catalyst was added in an amount of 0.5 wt %. Arising during the reaction ethyl and/or methyl alcohol was removed by distillation. After 3 h butyl esters of citric and succinic acids were main products of the conducted reaction. In the second stage of the synthesis, after reducing the pressure (to about 60–50 mbar), the temperature of the reaction mixture was gradually increased—at a rate of about 10 °C every 30 min until 180–190 °C. At this temperature, the reaction was continued; controlling changes in viscosity and analyzing ^1^H NMR spectra of the sampled reaction mixture. The process was carried out until the assumed molar mass of the product was obtained, which was dependent on the reaction time (Figure 1). After approximately 5–8 h number average molecular weights (*M*_n_) of produced polyesters in the range 5–8 × 10^3^ g/mol were obtained. All obtained products were dissolved in chloroform and precipitated in cold methanol and then dried in a vacuum dryer at 40 °C to a constant weight.

### 2.3. Synthesis of Poly(l-Lactide–co–Glycolide)–Block–Poly(Butylene Succinate-co-Butylene Citrate)

The obtained oligomers (Table 1) were used as a macroinitiator in the reaction of l-lactide polymerization or copolymerization of l-lactide with glycolide conducted in accordance with the ring opening polymerization (ROP) mechanism. Polymerizations were carried out in bulk at 120 °C for 5 days, using a low-toxic zirconium(IV) acetylacetonate catalyst in an amount consistent with a monomer/catalyst molar ratio of 1000/1. The same molar ratio of l-lactide to glycolide as 85:15 was used in all syntheses. The synthesis process was shown in the following example for the preparation of NL 20 copolymer.

Into a dry 0.25 dm^3^ round-bottomed flask fitted with connection to the argon source and to a vacuum pump, with mechanical stirrer, and heated on an oil bath, 0.425 mol (61.2 g) l-lactide and 0.075 mol (8.7 g) glycolide were added under protection of the argon cushion. After both monomers melting at 110 °C, stirring was started and then 30 g of NG 23 oligomer (Table 1) was added in portions. The reaction was carried out for about 5 days. All obtained copolymers were purified by dissolving in chloroform and then precipitation in cold methanol. Samples were dried to constant weight at room temperature under vacuum.

### 2.4. Measurements

#### 2.4.1. Nuclear Magnetic Resonance (NMR) Spectroscopy

The composition of the polymers was determined with NMR measurements. Based on the observation of amount of end groups on the ^1^H NMR spectrum, the number average molecular weight of these compounds was also determined. The ^1^H NMR spectra of the copolymers were recorded at 600 MHz with the Advance II Bruker Ultrashield Plus Spectrometer (Billerica, MA, USA) and with use a 5 mm sample tube. Deuterated chloroform was the solvent and as the internal standard tetramethylsilane was used. All ^1^H NMR spectra were obtained with 32 scans, a 2.65 s acquisition time and an 11 ms pulse at 26 °C. The ^13^C NMR spectra of the synthesized polymers were recorded at 150 MHz, using the same spectrometer and conditions as the proton spectra. The acquisition time was 0.9 s, the pulse width was 9.4 ms, the delay between pulses was 2 s and the spectral width was 36,000 Hz.

#### 2.4.2. Thermal Properties

By differential scanning calorimetry (DSC, DuPont 1090B apparatus calibrated with gallium and indium) thermal properties, such as glass transition temperatures and heats of melting and crystallization of obtained copolymers were examined. The glass transition temperature was determined with heating and cooling rate of 20 °C/min in the range between −100 and 220 °C, according to the ASTM E 1356-08 standard. 

#### 2.4.3. Fourier Transform Infrared (FTIR) Spectroscopy 

The spectra were recorded in KBr discs in the range of 4000–400 cm^−1^ at 64 scans of samples using JASCO FT/IR-6700 spectrophotometer (Easton, MD, USA) with a resolution of 2 cm^−1^.

#### 2.4.4. Measurement of Average Molecular Mass and Mass Dispersion

The average number of molecular mass (*M*_n_) and the mass dispersion coefficient (*Đ*) were determined by gel permeation chromatography (GPC) (Viscotek Rimax apparatus) in chloroform using calibration on polystyrene standards, at 25 °C with a flow of 1 mL/min, using two Viscotek 3580 columns (Malvern Panalytical Ltd., Malvern, United Kingdom) with a refractive detector. The phenomenon of the presence of high branched and high molecular structures was observed using GPC (Viscotek Rimax apparatus) in THF, 25 °C at a flow of 1 mL/min using infrared and viscosity detectors.

#### 2.4.5. Determination of the Amount of Active Hydroxyl Groups in Obtained Copolymers

Presence of hydroxyl groups in the obtained oligomers was initially estimated using comparative analysis of ^1^H NMR spectra before and after reaction with trichloracetyl isocyanate in deuterated chloroform solution. To determine the amount of hydroxyl groups in oligomers, the method described in ASTM D 2849-69 was used too, consisting of esterification of hydroxyl groups with phthalic anhydride in pyridine in the presence of imidazole as a catalyst and then hydrolysis of excess phthalic anhydride. The resulting acid groups were titrated with a sodium hydroxide solution in the presence of phenolphthalein. The average number of OH groups in the chain of individually synthesized copolymers were calculated. 

#### 2.4.6. Wettability and Surface Free Energy Tests 

Wettability and surface free energy (SFE) tests were performed on polymeric films prepared by dissolving 0.5 g of each polymer in 10 mL dichloromethane (DCM), pouring into a 9 cm diameter glass Petri dish and left to evaporate the solvent for 24 h. Tests were done on drop shape analysis system (DSA 25, Kruss, Germany) with the use of ultra-high quality water (UHQ-water produced in UHQ PS apparatus, Elga) using the sessile drop method. SFE was calculated according to Owens–Wendt equation using water and diiodomethane (Sigma Aldrich, Germany) as polar and non-polar liquids, respectively. In each case, 10 drops (0.5 μL in volume) were seeded on the surface of the samples and the contact angle was measured automatically. The results were presented as mean ± standard error of the mean (S.E.M). Statistical significance was assessed according to one-way-ANOVA with Fischer’s Least Significant Difference (LSD posthoc test with the use of Origin Pro 2020 (OriginLab Corp., Northampton, MA, USA).

#### 2.4.7. Biological Evaluation

Biological tests were carried out on MG-63 osteoblast-like cells (European Collection of Cell Cultures, Salisbury, UK). Cells were seeded on foils, which were made in the same way as described in 2.4.6. After solvent evaporation round samples with diameter suitable for 24-well cell culture plates (tissue culture polystyrene, TCPS, Nunclon) were cut with a hole punch. Before cell culture the samples were exposed to UV sterilization for 20 min. 1.5 × 10^4^ cells in 2 mL MEM Eagle medium (PAN BIOTECH, Aidenbach, Germany) supplemented by 10% FBS, 1% antibiotics (penicillin/streptomycin), 0.1% amino acids and 0.2% pyruvate (all reagents from PAA Laboratories GmbH, Pasching, Austria) were seeded on each sample. Cells cultures on TCPS acted as control. After 1, 3 and 7 days of culture metabolic activity was measured by Alamar Blue test (Sigma Aldrich Kit) and live/dead staining (calcein AM and propidium iodide mixture at 1:1 ratio; both at concentration 1 mg/mL). The tests were performed in triplicate, the results were presented as mean ± S.E.M. Statistical significance was assessed as described in Section 2.4.6.

## 3. Results and Discussion

### 3.1. Synthesis and Characterization of Macro-Initiators—Poly(Butylene Succinate–co–Butylene Citrate)

**Scheme 1:**
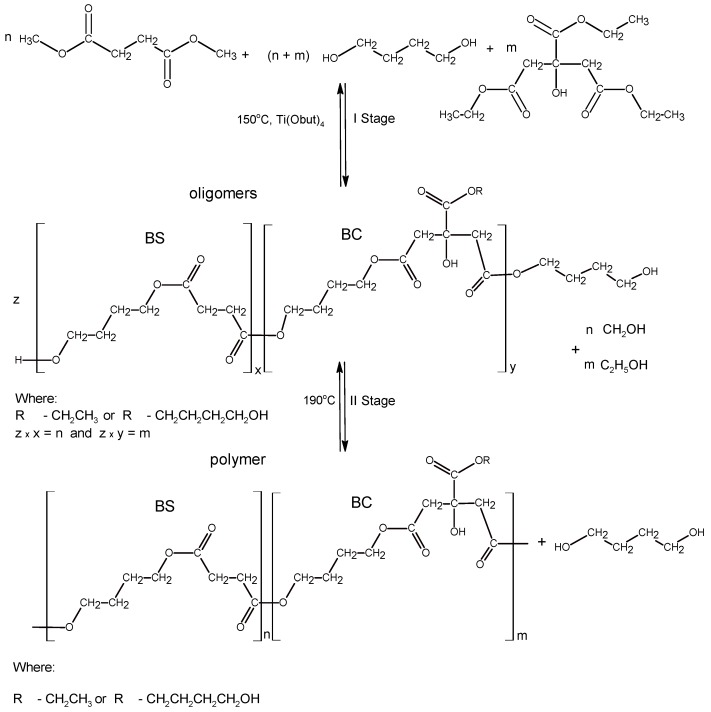
Preparation of polyester macroinitiator.

In the first stage of research, poly(butylene succinate–*co*–butylene citrate) and poly(butylene succinate) or poly(butylene citrate) were obtained by a typical polycondensation reaction carried out in bulk, according to Scheme 1. 

The aim of the synthesis was to obtain a series of polyesters, with an average molecular weight of 5–8 × 10^3^ g/mol, which in the next stage could be used as macro-initiators in the reaction of l-lactide polymerization or copolymerization of l-lactide with glycolide. The polymerization was carried out in accordance with the ROP mechanism. For this reason, the synthesized polyesters had to contain at least two active hydroxyl groups ended the chain. The application of citric acid derivatives as monomers should additionally allow introducing pendant hydroxyl groups into the manufactured copolymer chain. The process was carried out with a molar ratio of 1,4-butanediol to acid esters from 1.07 to 1 in the presence of a Ti(OC_4_H_9_)_4_ catalyst. It turned out that the above reaction conditions guaranteed obtaining non-crosslinked, soluble copolymers without a need to block the pendant hydroxyl groups of citrate ester. By changing the composition of the reaction mixture it is possible to obtain the oligoesters with different composition and molecular weights (Table 1).

The first phase of transesterification consisted of obtaining butanediol esters at a temperature of 150 °C; this stage lasted about 1 h. Then, after reducing the pressure in the reactor vessel, the temperature was gradually increased up to 180–200 °C. At this temperature, polycondensation was continued until the assumed molar mass was obtained (Figure 1).

**Figure 2:**
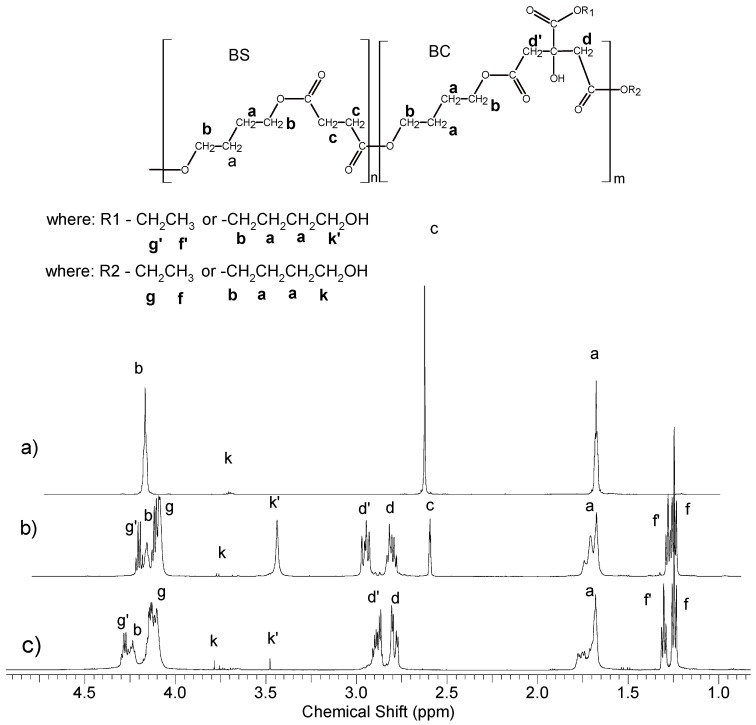
^1^H NMR spectra of polyesters (**a**) NG6 poly(butylene succinate); (**b**) NG 23 poly(butylene succinate–*co*–butylene citrate) and (**c**) NG48 poly(butylene citrate).

Finally, poly(butylene succinate) with a mass of 7.5–8.0 × 10^3^ g/mol was obtained after 6 h of reaction. In the case of obtaining poly(butylene citrate) with similar average mass, the reaction time was much longer and lasted about 13 h. Selected ^1^H NMR spectra of oligomers along with the signal assignments are shown in Figure 2. Based on the analysis of the signal intensity of the –CH_2_-OH end groups appearing in the ^1^H NMR spectrum at δ = 3.6 ppm (marked as k in Figure 2), their average molecular weight was determined. The composition of the oligomers did not differ significantly from the initial reaction mixture. Both, on proton and carbon spectra (Figures S1 and S2 in Supplementary Materials) there was a lack of signals associated with the methyl group of the starting succinate ester. However, it was different in the case of oligomers containing citrate units, and especially in the case of poly(butylene citrate). Based on ^1^H NMR spectra (Figure 2), it was found that 1/3 amount of the chain terminations still contains the starting ethyl ester groups (signals f and f’). Since these signals occur at various chemical shifts, they are both esters of the side groups as well as end groups in a quantity ratio of 1:2, respectively. Therefore, the chains growth runs in large part on the ester end groups of the starting ethyl citrate, but also on the side ester groups. The reason for this phenomenon is the non-stoichiometric amount of used 1,4 butanediol (Table 1). In the case of attempts to increase the amount of this diol the crosslinked insoluble product was obtained. 

**Figure 3:**
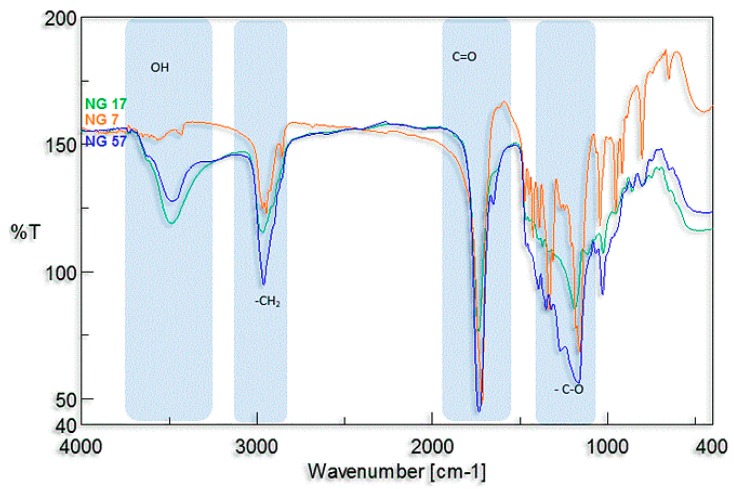
FTIR spectra of macroinitiators samples; NG 7 poly(butylene succinate), NG 17 poly(butylene succinate–*co*–butylene citrate) and NG 57 poly (butylene citrate).

For polymers containing butylene citrate units, it was necessary to clarify whether the hydroxyl side group of this compound is still present and active after polymerization. For this purpose, additional ^1^H NMR measurements of the obtained compounds after reaction with trichloroacetyl isocyanate were performed. The total shifts of the methyl signals of butylene citrate units –CH_2_C(OH)(COOR)CH_2_– (at δ = 2.81 ppm and δ = 2.87 ppm, signal d, d—Figure 2) in the direction of δ = 3.32 ppm and 3.25 ppm were observed (Figure S3 in Supplementary Materials). This phenomenon was caused by the reaction of the isocyanate group with active side hydroxyl groups, to form urethane bonds. This means that all side OH groups did not undergo changes during polymerization and remained active. Using the titration analysis, the amounts of OH groups in individual oligomers were estimated (Table 1). The obtained results confirmed the presence of active side hydroxyl groups too. The presence of these groups has also been confirmed with infrared spectroscopy. In the FTIR spectra, a distinct characteristic absorption band was observed in the range of 3300–3000 cm^−1^ corresponding to the stretching vibrations of hydroxyl groups (Figure 3). For poly(butylene succinate–*co*–butylene citrate) and poly(butylene citrate) the observed bands were much stronger than for poly(butylene succinate). The observed differences result from the existence of an additional side hydroxyl group in the butylene citrate units in the copolymer chain. The appearance of stronger band depends on the concentration of citrate units, which definitely confirms previous observations that hydroxyl groups of started ethyl citrate do not participate in the conducted polytransesterification.

The influence of the composition and chain structure of the obtained polymers on thermal properties was examined with DSC measurements. Poly(butyl succinate) samples showed the highest degree of crystallinity (fusion enthalpy ΔH about 80 J/g). With the increased content of butylene citrate units in the copolymer chain, the semi-crystallinity of such material gradually decreased (Table 1). Samples with content of above 50% mol citrate units were completely amorphous. The main reason for this phenomenon is the observed growth of the number of branched structures of chain, with an increase in the share of citrate units.

For poly(butylene succinate) samples, the GPC elugram (Figure S4 in Supplementary Materials, NG 6) shows relatively low dispersion of molecular weight. For the other polymers containing citrate units in the chain, their molecular mass dispersion significantly increased, however all these polymers were completely soluble.

### 3.2. Synthesis and Characterization of l-Lactide Copolymers with Butylene Succinate and Butylene Citrate

**Scheme 2:**
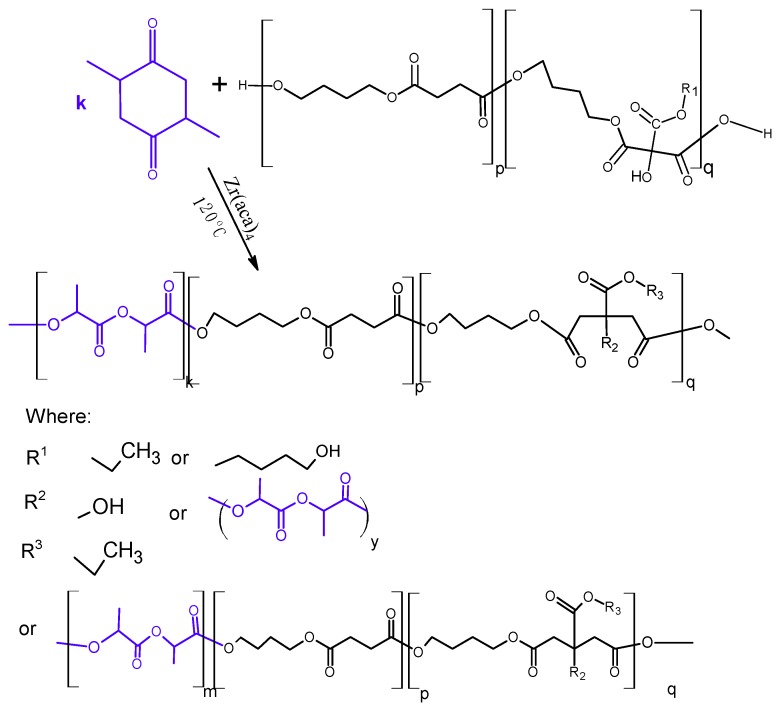
Polymerization of l-lactide using polyesters as a macroinitiator.

Then, the obtained polyesters (Table 1) were used as macroinitiators for the l-lactide polymerization, thus obtaining triblock copolymers (Scheme 2). Polymerization was carried out in bulk with using zirconium(IV) acetylacetonate as a catalyst, a compound previously tested as an effective, non-toxic catalyst or initiator of the ROP reaction of lactides and lactones [18,19]. The choice of this catalyst was also driven by the fact that the compound was active in the ROP polymerization even in the presence of organic acids [20]. So, the use of such a catalyst guarantees the possibility of conducting polymerization in the presence of compounds containing carboxylate or carboxyl groups, which may be the reaction by-products. 

During the polymerization of l-lactide in the presence of poly(butylene succinate), a high conversion of this monomer was obtained regardless of the composition of the starting reaction mixture (Table 2). The average molecular weights were close to the predicted. DSC tests (Figures S5 and S6 in Supplementary Materials) showed a high degree of crystallinity for all synthetized l-lactide copolymers. The copolymer containing 80% l-lactidyl units presented crystallinity regions associated with the arrangement of lactidyl blocks. These domains melted at 165 °C (Table 2, NG7). In the case of an increase amount of succinate units forming chain block, appearance of two melting enthalpies is observed. The one related to the melting of domains formed from butyl succinate is at 108 °C and the second derived from domains of lactidyl blocks melting at 150 °C (Figure S5b in Supplementary Materials) was observed. In turn in a copolymer with a low content of lactidyl at 108 °C we observed only one enthalpy of melting of areas formed by units of butylene succinate. In this case, the length of the lactidyl blocks was too small to be able to form crystalline areas.

Polymerization of l-lactide, carried out in the presence of macroinitiators containing both butylene succinate and butylene citrate units in the chain (polyesters NG 17 and NG 49) was significantly slower than previously. About 90% of conversion of l-lactide was achieved after 140 h (Figure S7 in Supplementary Materials). When the macroinitiator with low content of citrate units was used, the obtained l-lactide copolymer possessed a composition similar to the starting reaction mixture, and its molecular weight was slightly lower than the theoretical one (Table 3, NG20). 

**Figure 4:**
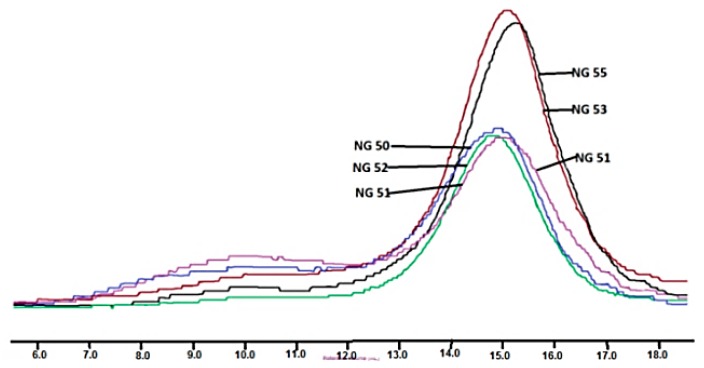
GPC elugrams of poly(l-lactide)–*block*–poly(butylene succinate–*co*–butylene citrate) and poly(l-lactide)–*block*–poly(butylene citrate).

However, when macroinitiators contained a significantly higher amount of citrate units the result was different. In this case, chloroform soluble terpolymers were obtained, but with unexpectedly high inherent viscosity, enormous weight average molecular weight and very high molecular mass dispersion (Table 3, NG 51, NG53 and NG55). The reason for this phenomenon was the existence of a high molecular mass phase (reaching several hundred thousand g/mol) observed on GPC elugram (Figure 4). 

**Figure 5:**
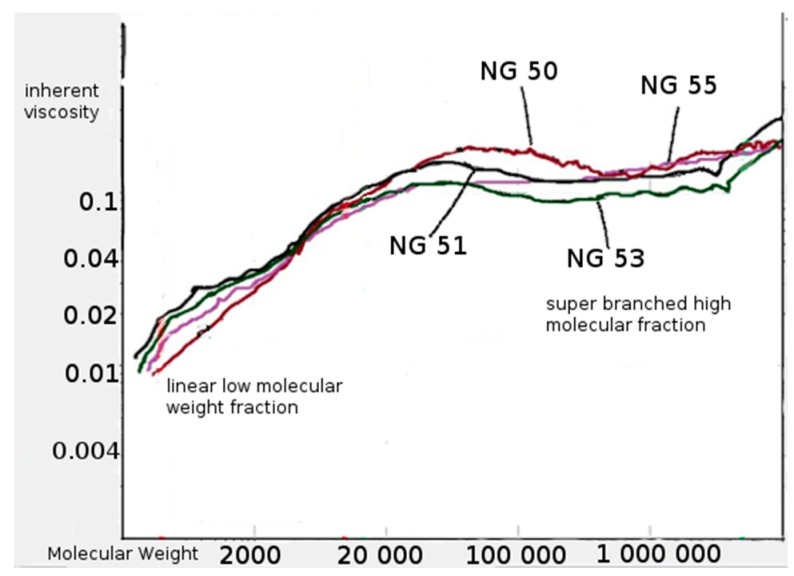
Relationship between the inherent viscosity and molecular weight of obtained poly(l-lactide)–*co*–poly(butylene citrate).

The supplementary GPC tests were carried out using an additional viscosity detector. The polymer fraction with very high *M*_w_, i.e. between 200 × 10^3^ g/mol and 2–3 × 10^6^ g/mol was found in the polymerization products. The solution of this fraction, regardless of molecular weight, presented practically the same intrinsic viscosity (Figure 5). 

**Scheme 3:**
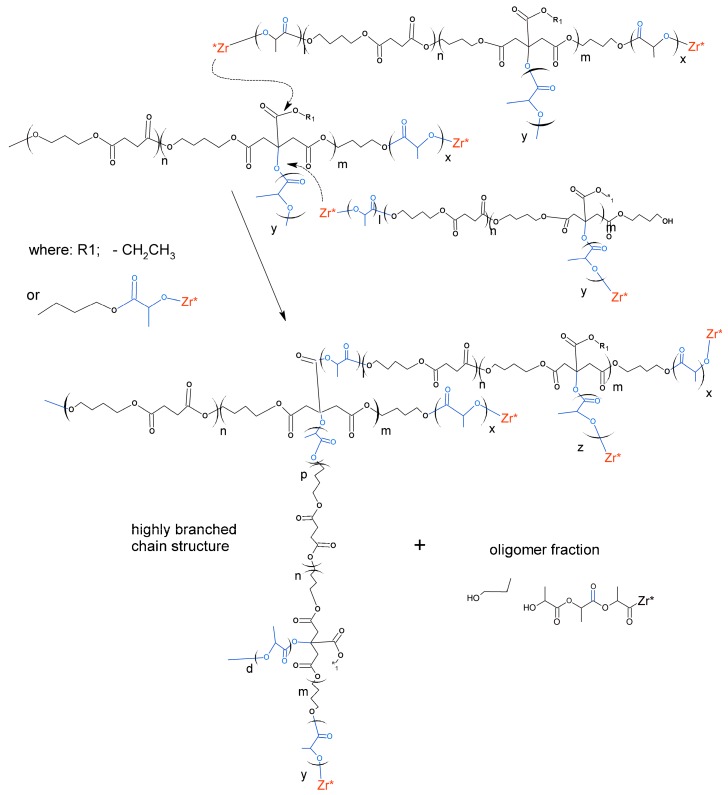
The course of intermolecular transesterification process causing the formation of chain branching structure.

The observed phenomenon indicates a specific, highly branched chain structure of polymers belonging to the discussed high molecular fraction of products [21]. The low molecular fraction with *M*_w_ from several hundred g/mol to about 150,000 g/mol showed compliance with the Mark–Houwink equation, so it was a fraction of linear chain copolyesters. The products with a very high mass and highly branched chain structure are formed as a result of intermolecular transesterification that occurs in parallel with the main chain growth reaction. This process arrives as a result of attacks of the active ends of the growing chain on the ester groups of the side lactidyl chains formed on the tertiary hydroxyl groups of the citrate units. For the above reason, the intensity of discussed transesterification depends on the content of citrate units in the chain of used macroinitiator. As a result of the transesterification, the molecules merge with each other, resulting in a rapid increase in the mass of associated molecules and the formation of complex branched chain structures. At the same time, short linear oligomers of polylactide and oligomers containing ethyl ester group are created as illustrated in Scheme 3.

*M*_w_ from several hundred g/mol to about 150,000 g/mol showed compliance with the Mark–Houwink equation, so it was a fraction of linear chain copolyesters. The products with a very high mass and highly branched chain structure are formed as a result of intermolecular transesterification that occurs in parallel with the main chain growth reaction. This process arrives as a result of attacks of the active ends of the growing chain on the ester groups of the side lactidyl chains formed on the tertiary hydroxyl groups of the citrate units. For the above reason, the intensity of discussed transesterification depends on the content of citrate units in the chain of used macroinitiator. As a result of the transesterification, the molecules merge with each other, resulting in a rapid increase in the mass of associated molecules and the formation of complex branched chain structures. At the same time, short linear oligomers of polylactide and oligomers containing ethyl ester group are created as illustrated in Scheme 3.

**Figure 6:**
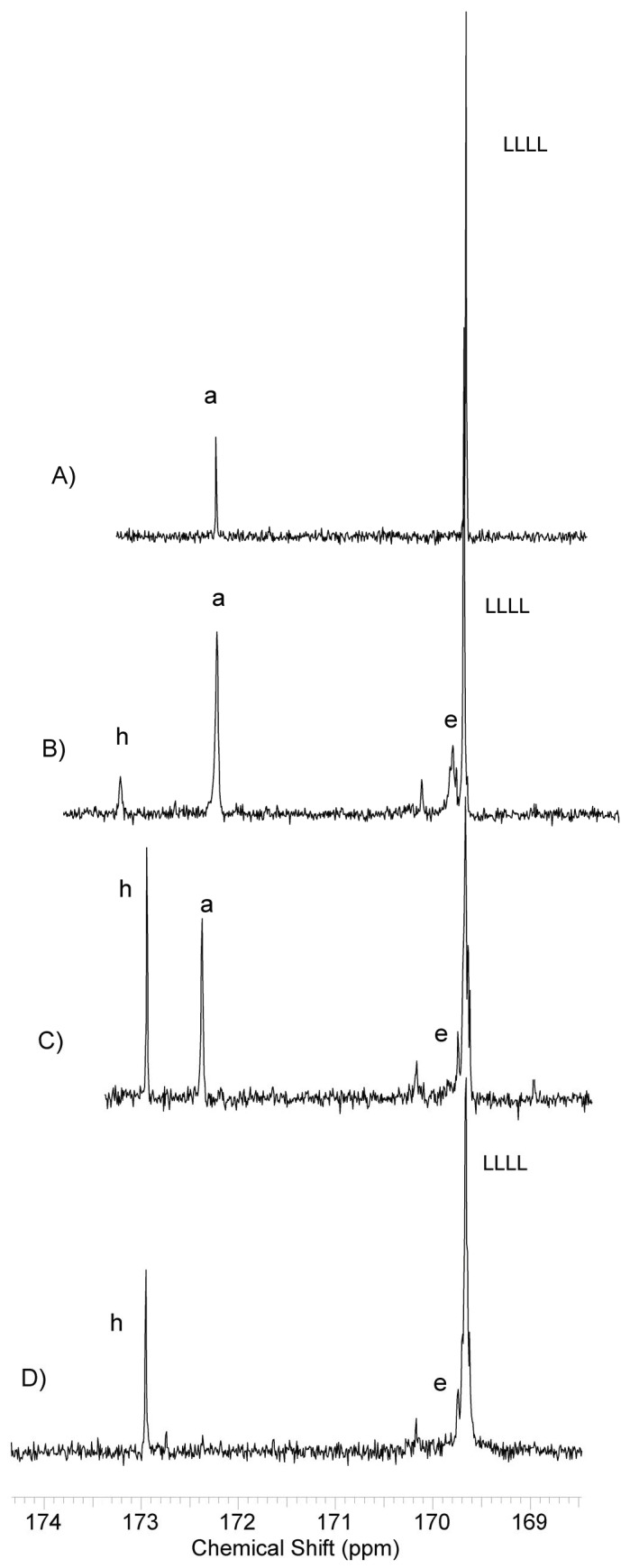
^13^C NMR spectra (carbonyl region, in CDCl_3_) of (**A**) NG7—poly(l-lactide)–*block*–poly(butylene succinate), (**B**) NG 20—poly(l-lactide)-*block*-poly(butylene succinate–*co*–butylene citrate), (**C**) NG51—poly(l-lactide)–*block*–poly(butylene succinate–*co*–butylene citrate) and (**D**) NG53—poly(l-lactide)–*block*–poly(butylene citrate). Where; h, e—signal carbonyl carbons of butyl citric units, a—signal of carbonyl carbons of butyl succinic units, LLLL—signal of carbonyl carbons of lactidyl units.

Previously we described a very similar and analogical phenomenon, which proceeded during the polymerization of cyclic carbonates containing side ester groups [22]. A mechanism of transesterification in which the attack of active ends is directed to the ester bonds of the main chain is also very probable. However, in the ^13^C NMR spectra, there are no signals that may originate from hypothetically formed on this way lactidyl-citrate or lactidyl-succinate sequences (Figure 6).

The carbonyl region signals of the citrate and succinate units are not split. Comparing the spectra of samples containing citrate units (Figure 6D) with the analogue containing only lactidyl block and butyl succinate, it can be seen that the apparent LLLL signal split is rather an effect of overlapping with a signal assigned to carbon of citrate carbonyl. However, an attack of active lactide ends of the growing chain on ester bonds of lactidyl blocks of adjacent molecules is possible. Such transesterification can lead to the formation of multi-block copolymers and shorter active lactidyl chains without visible changes in observed NMR spectra. Therefore, for all obtained copolymers it is impossible to definitely say that they have a three-block structure. 

**Figure 7:**
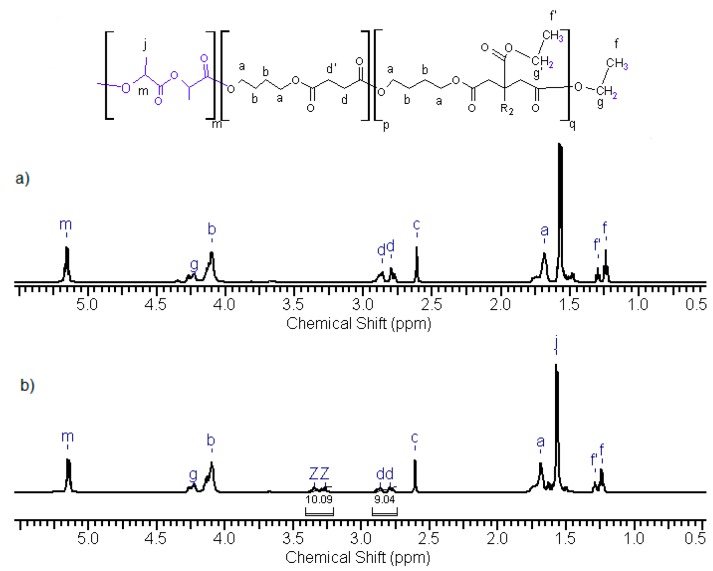
^1^H NMR spectrum of poly(l-lactide)–*block*–poly(butylene citrate–*co*–butylene succinate) samples; (**a**) before reaction with trichloracetyl isocyanate and (**b**) after reaction with trichloracetyl isocyanate.

The composition of the final copolymers was determined on the basis of ^1^H NMR spectra analysis. Figure 7 shows as example selected proton NMR spectra of poly (l-lactide)–*block*–poly(butylene succinate–*co*–butylene citrate). In this spectrum, signals in the range δ = 5.18 ppm (m, 4H) and 1.6 ppm (j, 6H) are derived from protons of lactidyl units; –CH– and CH_3_ groups. Other signals are related to the presence of citrate and succinate units derived from the macroinitiator—poly (butylene succinate–*co*–butylene citrate). These signals are the same as it was previously observed in the spectrum of macroinitiator before the lactide polymerization. In order to check the possible presence of active side hydroxyl groups, the additional ^1^H NMR analysis was performed after reaction of the samples of copolyesters with trifluoroacetyl isocyanate. A shift of a part of the signals of the methylene protons –CH_2_ (OH)(COOR) CH_2_– (citrate units) was detected; from the initial δ = 2.81–2.87 ppm (Figure 7a, denoted as d, d) to δ = 3.25–3.32ppm (Figure 7b marked as Z, Z). Based on the resulting changes, the content of active side hydroxyl groups in the chain of obtained copolymers was estimated, which in most of the tested samples was about 50%–60% of the number of starting groups. Thus, during the polymerization, only about half of these groups reacted with lactide, forming side polylactide chains. These chains have been involved in observed intermolecular transesterification reactions discussed earlier.

Analysis of DSC curves of the obtained copolyesters shows a strong dependence of their composition on the thermal properties. NG 20 copolymer containing 55% mol of lactidyl groups and 29% succinate units, is characterized by the presence of two enthalpies of melting (Table 3), one associated with ordered lactidyl domains (at 115 °C) and second derived from succinate blocks (at 40 °C). As the amount of citrate units in the chain increases (at the expense of succinate units, Table 3, NG 51) only enthalpy of melting of organized lactidyl structures is observed. In contrast, copolymers made of l-lactide and butylene citrate blocks, i.e., NG 53 and NG 55, are completely amorphous. The glass transition temperature drops strongly as the amount of butyl citrate units in the copolymer chain increases. The observed phenomenon is associated with the increase in the branched chain structures of these copolymers.

### 3.3. Synthesis and Characterization of Poly (l-lactide–co–glycolide)–block–poly(butylene succinate–co–butylene citrate)

Due to the relatively high crystallinity of the previously described copolymers containing longer l-lactide blocks, attempts were made to obtain analogous copolymers but with lower, containing the chain blocks made of l-lactide and glycolide random sequences. Such terpolymers appear to be better as a potential material for forming scaffolds. The terpolymers were successfully obtained by copolymerization of l-lactide and glycolide carried out in the presence of poly(butylene succinate–*co*–butylene citrate) with an average molecular weight *M*_n_ of about 5000 g/mol, where the molar ratio of BS units to BC was as: 30:70 or 50:50 (Table 4, Scheme S1 in Supplementary Materials). Similarly to the previously described polymerization of l-lactide, after about 6 days a high conversion of monomers was obtained.

**Figure 8:**
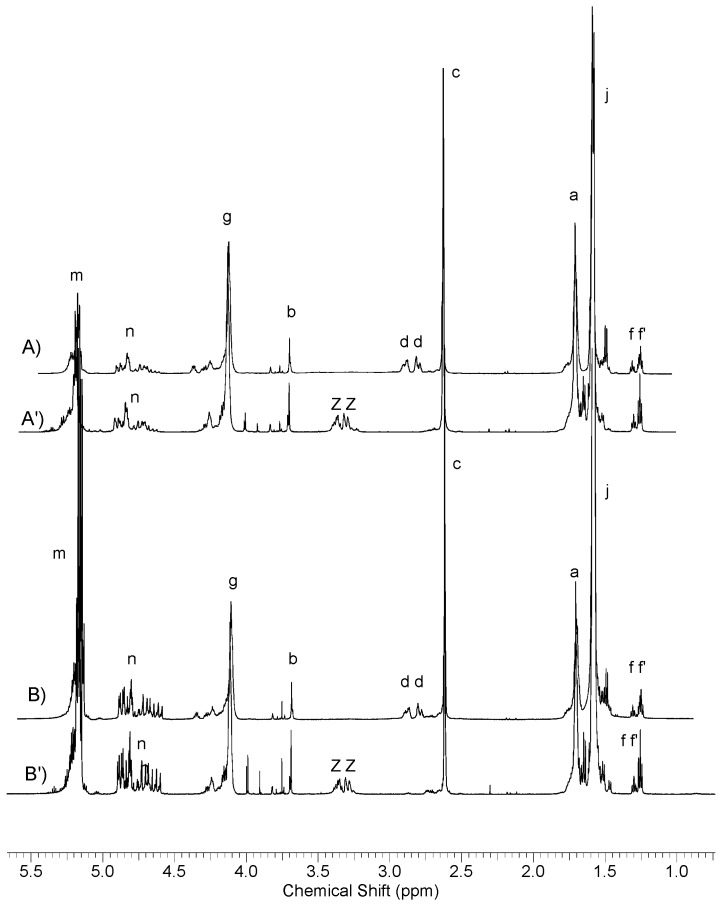
Spectra ^1^H NMR poly(l-lactide–*co*–glycolide)–*block*–poly(butylene succinate–*co*–butylene citrate); (**A**) sample NL13, (**A’**) sample NL13 after reaction with trichloroacetyl isocyanate, (**B**) sample NL14 and (**B’**) sample NL14 after reaction with trichloroacetyl isocyanate.

Based on the obtained ^1^H NMR spectra (Figure 8), the actual copolymer composition and monomer conversion were determined (Table 4). The proton spectra differed from those described earlier only by the presence of an additional signal of glycolyl methylene protons (Figure 8, signal n). Quite unexpectedly, even with relatively high contents of citrate units in the copolymer chain, the phenomenon of formation of a highly branched fraction was not observed (Table 4, NL20, NL 12–13). In the obtained copolymers virtually all OH groups present at tertiary carbon in citrate units of the chain were active in the reaction with isocyanate. After reaction of the resulting product with trichloroacetyl isocyanate the signals of methylene groups of citric acid derivative—dd were completely shifted to signals ZZ. This means that side hydroxyls did not participate in the copolymerization reaction and forming side chains. So, forming high molecular fractions according to transesterification mechanism presented on Scheme 3 was rather impossible.

**Figure 9:**
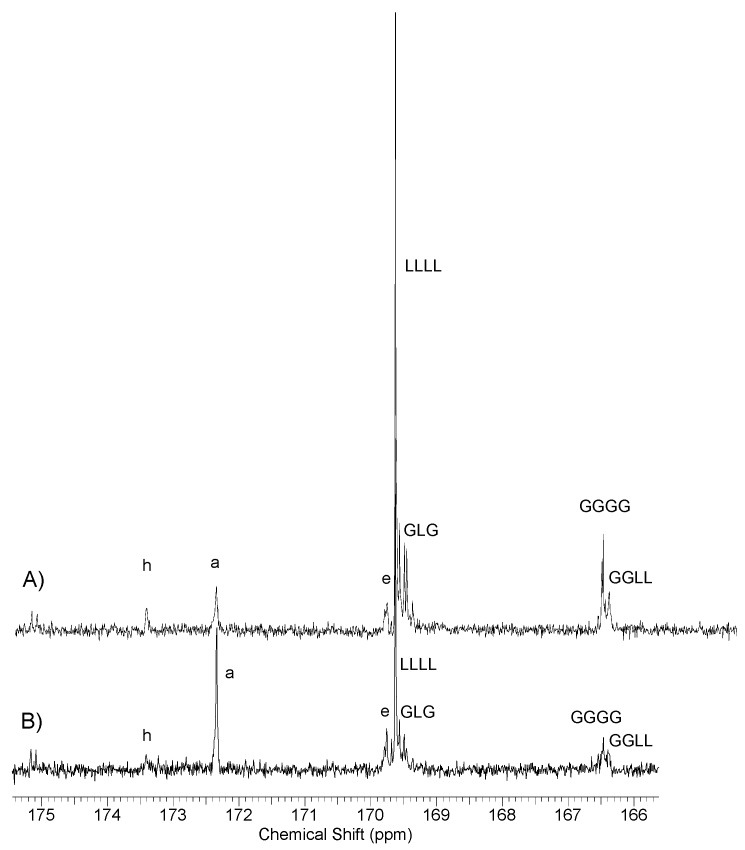
Spectra ^13^C NMR samples of poly(l-lactide–*co*–glycolide)–*block*–poly(butylene succinate–*co*–butylene citrate); (**A**) sample NL15 and (**B**) sample NL 13.

Analysis of ^13^C NMR spectra in the range of carbonyl carbons showed the presence of the same signals as in the case of the previously presented l-lactide/butyl succinate/butyl citrate copolymers (Figure 9). Main signals are derived from the carbons of succinate (signal a) and citrate units (signals e and h) as well as lactidyl (LLLL) and additionally glycolide (GGGG or GGLL). Only the carbon signal LLLL was clearly split, as well as the signal associated with the presence of glycolidyl units (GGGG and GGLL signals), analogically as it was the case of poly(l-lactide–*co*–glycolide) or poly(lactide–*co*–glycolide)–*block*–poly(ethylene glycol) copolymers [18,23]. The absence of additional signals that could be derived from shorter chain sequences suggests that the chains of received copolymers have a block chain structure.

In the DSC thermograms of these copolymers presence of two distinct melting enthalpies associated with ordered structured areas were observed; the larger built with the succinate units blocks (at 78–105 °C) and the much smaller created with the lactidyl units blocks (at 113–139 °C). Compared to previously synthesized l-lactide/butyl succinate/butyl citrate copolymers, there is a significant decrease in the crystallinity of lactidyl domains, which is associated with the presence of the glycolyl-lactidyl sequence in the chain, causing a shortening of the length of lactidyl blocks. The glass transition temperatures are clearly higher, which is mainly due to a practically lack of copolymer fraction with a strong branched structure of the chain (Table 4).

### 3.4. Wettability and Surface Free Energy

**Figure 10:**
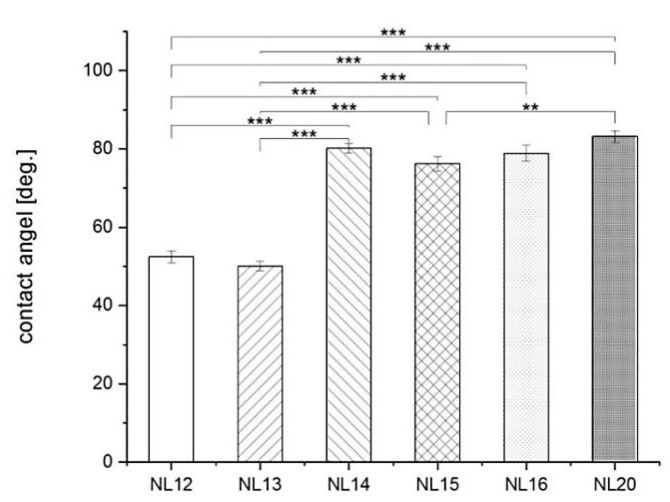
Water contact angle results of different polymers; mean ± S.E.M., n = 10. Asterisks show statistical significance at p** < 0.01, p*** < 0.001.

**Figure 11:**
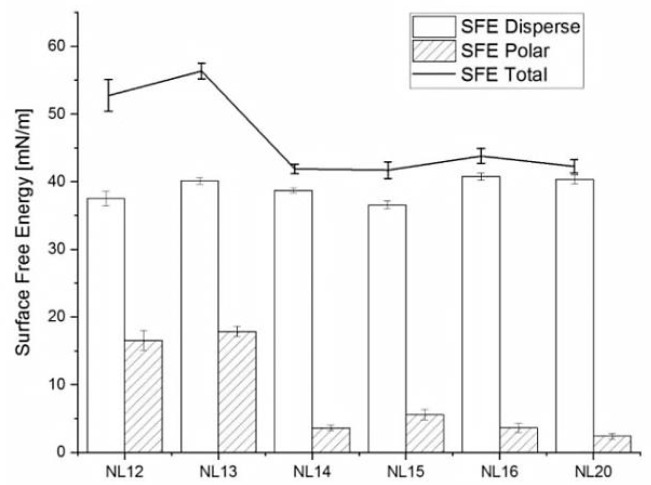
Surface free energy values: total, dispersive part (white boxes), and polar part (striped boxes); mean ± S.E.M., n = 10.

Results of wettability (Figure 10) show that NL12 and NL13 samples are the most hydrophilic (water contact angle 51.3° and 50.1°, respectively) as compared to all other samples with a contact angle of about 80°. The highest surface free energy SFE had the samples with the lowest wettability, i.e., NL12 and NL13 (Figure 11). The results show that there is not much difference in the SFE dispersion component as compared to the polar component. Materials that are more hydrophobic have much lower polar part of SFE (NL14, NL15, NL16 and NL20) than those that are more hydrophilic (NL12 and NL13). 

### 3.5. Cell Metabolic Activity, Viability and Morphology 

**Figure 12:**
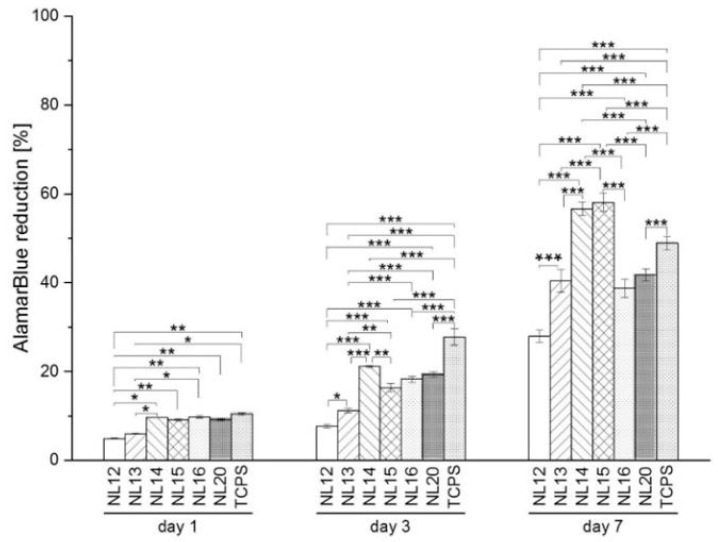
Metabolic activity of MG-63 cells cultured on different polymers and control TCPS measured by AlamarBlue test. Asterisks show statistical significance made with Fisher’s LSD test; p* < 0.05, p** < 0.01, p*** < 0.001.

Biological evaluation shows that MG-63 cells adhere and proliferate well on all the samples (Figure 12). On day 1 significantly lower cell viability was found on NL12 and NL13 samples as compared to all other samples and control TCPS. More differences were observed after 3 days of culture. The highest cell viability was found for NL14 but also on NL16 and NL20; there was no significant differences between those samples. It is worth emphasizing that after 7 days of cell culture, more cells were observed on NL14 and NL15 samples than on the control TCPS. Cell viability on NL14 and NL15 was similar and significantly higher than for other samples. 

**Figure 13:**
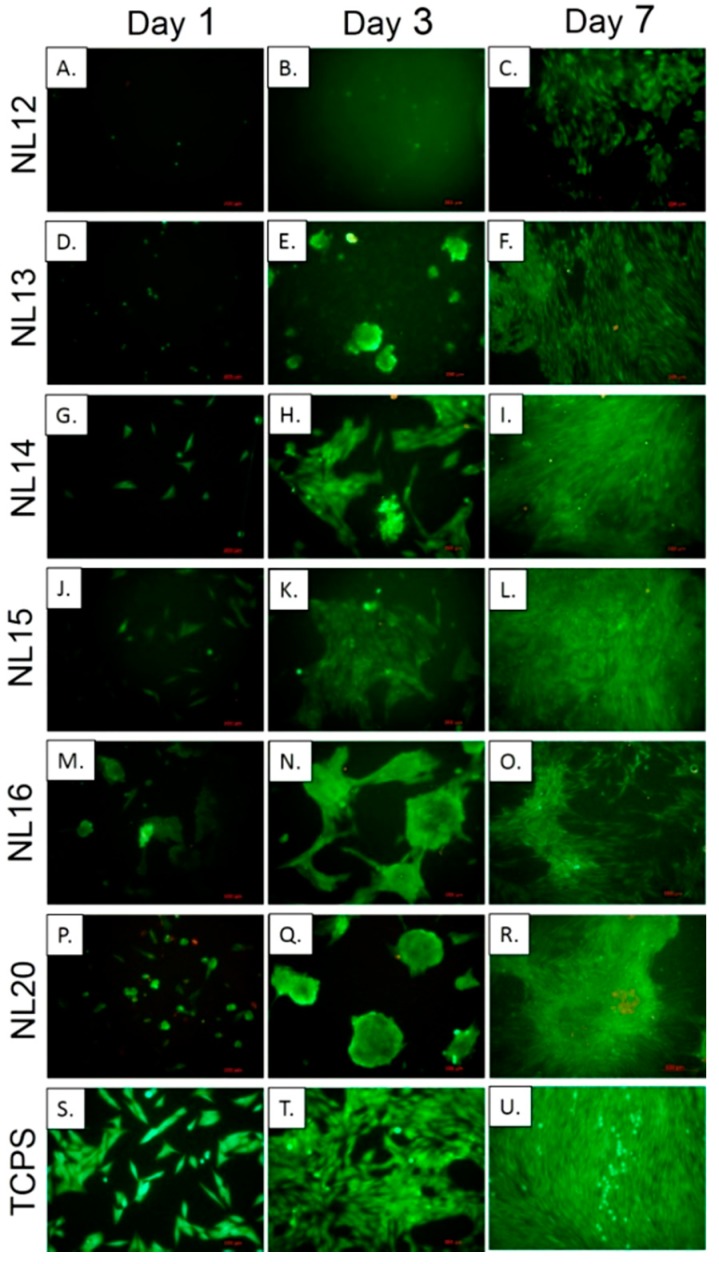
MG-63 cell morphology cultured for 1, 3 and 7 days on different polymers and controlled GTCPS, staining with calcein AM and propidium iodide; green—live cell, red—dead cell.

Figure 13 shows cell morphology after live-dead staining. After day 1, cells on NL14 (Figure 13G) and NL15 (Figure 13J) had similar morphology as on control TCPS (Figure S13 in Supplementary Materials); i.e. they were well spread and had polygonal shape. On other materials (Figure 13A,D,M,P), the cells were more round and not very well spread. On the following days of cell culture, especially on day 3, the cells tended to aggregate (Figure 13E,N,Q). Exceptions were samples NL14 and NL15 (Figure 13H,K). After 7 days cell aggregates were observed on NL20 (Figure 13R); on the rest of the samples cells were homogeneously spread. What is more during all cell culture periods number of dead cells was very low (below 2%) and comparable to that observed on control TCPS. 

## 4. Conclusions 

Through the ROP polymerization of l-lactide and copolymerization of l-lactide with glycolide carried out in the presence of hydroxyls ended macroinitiators, a wide range of bioresorbable block copolymers was obtained. First, the macroinitiators were obtained by the polytransesterification reaction of succinic and citric acid esters and 1,4-butandiol. The properties of the obtained copolymers can be shaped in a wide range both by the composition of the polymerization mixture and by the composition and molecular weight of the macroinitiators used. The used method of synthesis allows relatively easily the introduction side, chemically active hydroxyl groups into the main chain of the final copolymers, without the need to block the hydroxyl group present at the quaternary carbon of citric acid esters. During the polymerization of l-lactide conducted with presence of polyesters containing butylene citrate units in the chain, obtained product held a fraction of very highly branched copolymers of very high molecular weight. The reason for this phenomenon is strong intermolecular transesterification directed to lactidyl side chains, formed as a result of chain growth on hydroxyl groups related to the quaternary carbon atoms of the citrate units. The most promising as a material for forming scaffolds seem to be the family poly(l-lactide–*co*–glycolide)–*block*–poly(butylene succinate–*co*–butylene citrate), especially those copolymers containing more than 60% mol of lactidyl. The latter had a relatively low molecular weight distribution unlike poly(l-lactide)–*block*–poly(butylene succinate–*co*–butylene citrate)s. The lack of polylactide chain growth on hydroxyl side groups prevented the occurrence of strong intermolecular transesterification, which was observed in previous lactide copolymer and which was responsible for the formation of high and low molecular polymeric fractions. 

Based on NMR studies, both at the stage of macroinitiators formation (Figure S2 in Supplementary Materials) and during ROP polymerization (Figure 6 and Figure 9), the carbonyl carbon signals of butyl citrate and butyl succinate units are not split. It is therefore difficult to show the formation of a significant amount of alternating chain sequences. However, intermolecular transesterification with an attack of active lactide ends of the growing chain on ester bonds of lactidyl blocks of adjacent molecules is possible. Such transesterification can leads to the formation of multi-block copolymers and shorter active lactidyl chains without visible changes on the observed NMR spectra. Therefore, for all obtained copolymers it is impossible to definitely say that they have a three-block structure.

Further research should focus on optimizing the composition of the copolymers and their chain structure, to adjust the properties of the obtained material according to the intended use (soft tissue or bone tissue scaffolds). The obtained polymers can also be an interesting carrier of drugs or other biologically active compounds that can be attached by means of OH side groups.

## Figures and Tables

**Table 1:** Properties of synthesized poly(butylene succinate), poly(butylene succinate–*co*–butylene citrate) and poly(butylene citrate).

Sample	SM:CE:BD	BS:BC (% mol)	*M*_n_ (g/mol)	*Đ*	N_OH_	*T*_g_ (°C)	*T*_m_ (°C)	Δ*H* (J/g)
NG 6	1:0:1.07	100:0	7000	2.2	≈2	−18	114	81
NG 17	7:3:10.7	68:32	7000	4.0	10	−33	77	52
NG 23	5:5:10.7	52:48	8000	8.7	17	−29	55	14
NG 49	3:7:10.7	27:73	7000	9.2	20	−29	-	-
NG 48	0:1:1.07	0:100	7500	10.5	24	−31	-	-

Where: SM:CE:BD—the molar ratio of methyl ester of succinic acid to ethyl ester of citric acid and to used 1,4 butanediol in the starting reaction mixture, BS:BC—the molar ratio of succinate units to citrate units in the copolymer chain, *M*_n_—average molecular weight calculated from ^1^H NMR spectra, *Đ*—molecular weight dispersion determined by GPC chromatography, *N*_OH_—number of OH groups in the oligomer molecule estimated on the basis of analytical tests carried out in accordance with ASTM D 2849-69, *T*_g_—glass transition temperature, *T*_m_—melting point of the crystalline phase, Δ*H*—enthalpy of melting the crystalline phase.

**Table 2:** Properties of obtained poly(l-lactide)–*block*–poly(butylene succinate) copolymers.

Sample	*C* (%)	M/I	(LA:PBS)^0^ (wt %)	LA:BS (wt %)	LA:BS (%mol.)	*M*_w_ (g/mol)	^T^*M*_w_ (g/mol)	*Đ*	*T*_g_ (°C)	*T*_m_ (°C)	Δ*H* (J/g)	η_inh_ (dL/g)
NG7	99	190/1	75:25	75:25	80:20	56; 200	68; 720	2.0	38	165	43	1.1
NG8	96	75/1	60:40	59:41	65:35	38; 100	44; 500	2.5	7	108; 150	29; 23	0.8
NG9	95	33/1	40:60	38:62	45:55	44; 500	43; 480	3.7	−16	108	50	0.8

Polymerization carried out in bulk at 120 °C for 120 h, catalyst Zr(acac)_4_ at a monomer/catalyst molar ratio as 1000/1, where: *C*—l-lactide conversion, M/I—monomer to initiator molar ratio (macroinitiator NG 6, poly(butylene succinate) with M_n_ about 7000 g/mol), (LA:PBS)^0^—weight ratio of l-lactide to poly(butylene succinate) in started reaction mixture, LA:BS—weight (and molar) ratio of lactidyl units to butylene succinate units in the copolymers chain, *M*_w_—weight average molecular mass of the copolymer determined in GPC measurements, ^T^*M*_w_—calculated theoretical average molecular weight of the copolymer, *Đ*—molecular weight dispersion determined in GPC measurements, *T*_g_—glass transition temperature, *T*_m_—temperature of melting of the crystalline phase, Δ*H*—enthalpy of melting of the crystalline phase, η_inh_—inherent viscosity of the copolymer in chloroform solution.

**Table 3:** Properties of the obtained poly(l-lactide)–*block*–poly(butylene succinate–*co*–butylene citrate) and poly(l-lactide)–*block*–poly(butylene citrate) copolymers.

Sample	Initiator	*C* (%)	M/I	(LA:BS:BC)^0^ (%mol.)	LA:BS:BC (%mol.)	*M*_w_ (g/mol)	*Đ*	*T*_g_ (C°)	*T*_m_ (C°)	Δ*H* (J/g)	η_inh_ (dL/g)
NG 20	NG 17	99	60/1	55:30:15	55:29:16	24; 200	2.2	11	40; 115	38; 6.1	0.3
NG 51	NG 49	88	60/1	55:12:23	49:14:27	190; 000	≈100	−1	126	16	1.3
NG 53	NG 48	96	75/1	59:0:41	60:0:40	203; 000	≈57	−15	-	-	1.7
NG 55	NG 48	98	12/1	19:0:81	19:0:81	148; 000	≈58	−26	-	-	1.6

Polymerization carried out in bulk at 120 °C, catalyst Zr(acac)_4_ with the monomers/catalyst molar ratio as 1000/1, Where: *C*—lactide conversion, M/I—molar ratio of monomers to initiator, (LA:BC:BS)^0^, LA: BC: BS—molar ratio of lactide to citrate and succinate units in the starting mixture, in the copolymer, *M*_w_—weight average molecular mass of the copolymer determined with GPC measurements, *Đ*—molecular mass dispersion, *T*_g_—glass transition temperature, *T*_m_—melting point of the crystalline phase, Δ*H*—enthalpy of melting the crystalline phase, η_inh—_inherent viscosity of the copolymer in chloroform solution.

**Table 4:** Characteristics of synthesized poly (l-lactide–*co*–glycolide)–*block*–(butylene succinate–*co*– butylene citrate).

Sample	Initiator	*C* (%)	M/I	(LA:GL:BS:BC)^0^ (% mol)	La:GL:BS:BC (% mol)	*M*_w_ (g/mol)	*Đ*	*T*_g_ (°C)	*T*_m_ (°C)	Δ*H* (J/g)	η_inh_ (dL/g)
NL 14	NL7	98	120/1	67:11:7:15	66:10:9:15	42; 500	3.5	32	78; 131	3.9, 2.0	0.45
NL 15	NL3	97	120/1	67:11:11:11	64:12:12:12	34; 300	3.7	33	83; 120	5.4, 1.5	0.41
NL20	NL7	97	60/1	55:9:11:26	57:10:11:22	28; 100	3.8	21	73; 117	14.2, 2.8	0.37
NL16	NL3	98	60/1	55:9:18:18	56:10:17:17	29; 400	3.8	23	77; 113	13.7, 0,9	0.35
NL12	NL7	91	40/1	46:7:14:33	47:8:13:32	15; 300	4.0	5	95; 139	15.4, 0,9	0.24
NL13	NL3	94	40/1	46:7:23:24	48:8:22:22	19; 700	3.9	6	105	17.0	0.28

Copolymerization of l-lactide (85% mol) and glycolide (15% mol) was carried out in bulk at 120 °C, for 6 days, with using catalyst Zr(acac)_4_ with both monomer/catalyst molar ratio as 1000/1, with macro initiators NL3—poly(BS 48%–*co*–BC 52% with *M*_n_ = 5000 g/mol or with NL7—poly(BS 30%–*co*–BC 70%), with *M*_n_ about 5000 g/mol, Where: *C*—monomers conversion, M/I—molar ratio of both monomers to macroinitiator, (LA:GL:BS: BC)^0^—molar ratio of l-lactide, glycolide, butyl succinic and butyl citric units in the reaction mixture, La:GL:BS:BC—molar ratio of lactidyl, glycolide, succinic and citric units, *Đ*—molecular weight dispersion, *T*_g_—glass transition temperature, *T*_m_—melting temperature of the crystalline phase, η_inh_—inherent viscosity of the copolymers in a chloroform solution at 25 °C.

## Supplementary Materials

The following are available online at https://www.mdpi.com/2073-4360/12/1/214/s1, Figure S1: ^13^C NMR spectrum of poly(butylene succinate)—polymer NG6, Figure S2: ^13^C NMR spectra of poly(butylene succinate–*co*–butylene citrate)—polymer NG48, Figure S3: ^1^H spectra of poly(butylene citrate) before reaction with trichloroacetyl isocyanate and after reaction, Figure S4: GPC elugrams of the synthesized butylene succinate/butylene citrate copolymers, Figure S5: DSC thermograms of poly(l-lactide)-*block*-poly(butylene succinate) - I run a) NG7, b) NG8 and c) NG9 copolymers, Figure S6: DSC thermograms of poly(l-lactide)-*block*-poly(butylene succinate)—II run a) NG7, b) NG8, c) NG 9 copolymers, Figure S7: Dependence of l-lactide conversion and reaction time during l-lactide polymerization conducted in the presence of poly(butylene citrate)—NG48 initiator, Figure S8: DSC thermograms of poly(l-lactide)-*block*-poly(butylene succinate–*co*–butylene citrate)—1^st^ run, sample; a) NG 51, b) NG 53, c) NG 55, Figure S9: DSC thermograms of poly(l-lactide)-*block*-poly(butylene succinate–*co*–butylene citrate)—2^nd^ run, sample; a) NG 51, b) NG 53, c) NG 55, Schema S1: Course of copolymerization of l-lactide with glycolide with use of poly(butylene succinate–*co*–butylene citrate) as reaction macroinitiator.
