# Supplementary material for: Synthesis and Properties of Bioresorbable Block Copolymers of l-Lactide, Glycolide, Butyl Succinate and Butyl Citrate
Source: Polymers (Basel). 2020 Jan 15;12(1):214. doi: 10.3390/polym12010214 (PMC7023550; doi:10.3390/polym12010214)
Supplement: Supplementary file 1 [file polymers-12-00214-s001.pdf]

Article

Synthesis and Properties of Bioresorbable Block Copolymers of L-Lactide, Glycolide, Butyl Succinate and Butyl Citrate

Natalia Śmigiel-Gac[1], Elżbieta Pamuła[2], Małgorzata Krok-Borkowicz[2], Anna Smola-Dmochowska[1] and Piotr Dobrzyński*[1],

[1] Centre of Polymer and Carbon Materials, Polish Academy of Sciences Zabrze; ngac@cmpw-pan.edu.pl, asmola@cmpw-pan.edu.pl, pdobrzynski@cmpw-pan.edu.pl

[2] AGH University of Science and Technology, Faculty of Materials Science and Ceramics, Al. Mickiewicza 30, 30-059 Kraków, Poland; epamula@agh.edu.pl, krok@agh.edu.pl

* Correspondence: pdobrzynski@cmpw-pan.edu.pl

Supplementary information

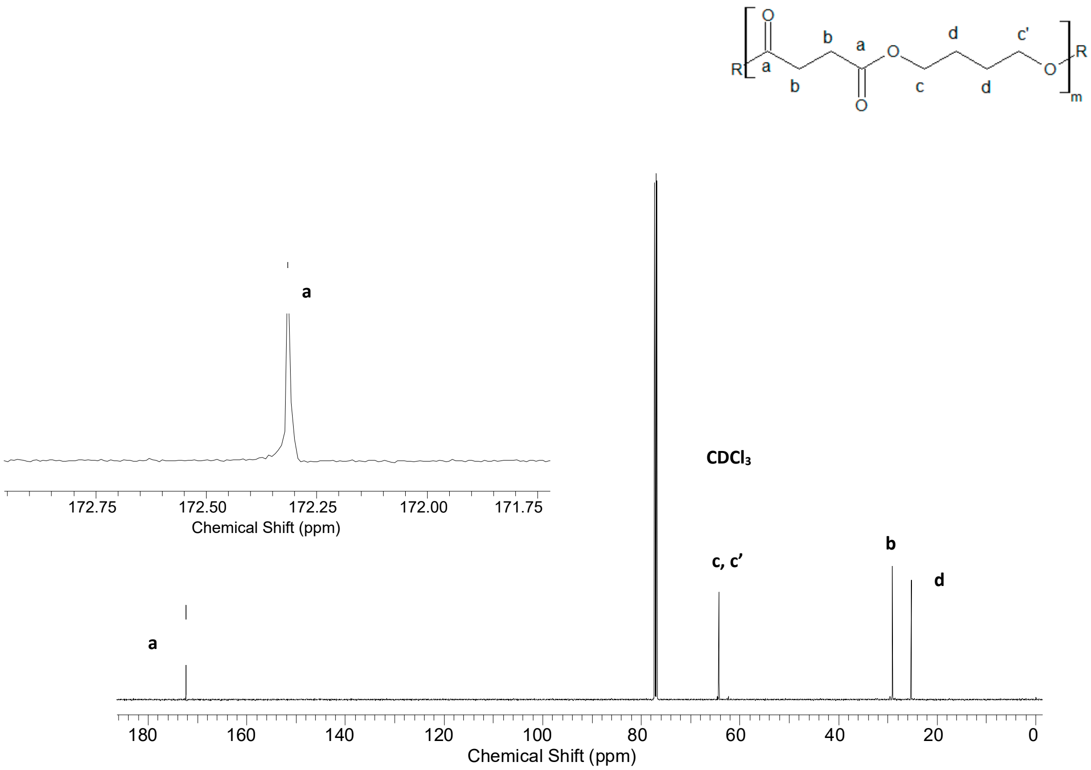

Figure S1 . ^{13}C NMR spectrum of poly(butylene succinate) – polymer NG6

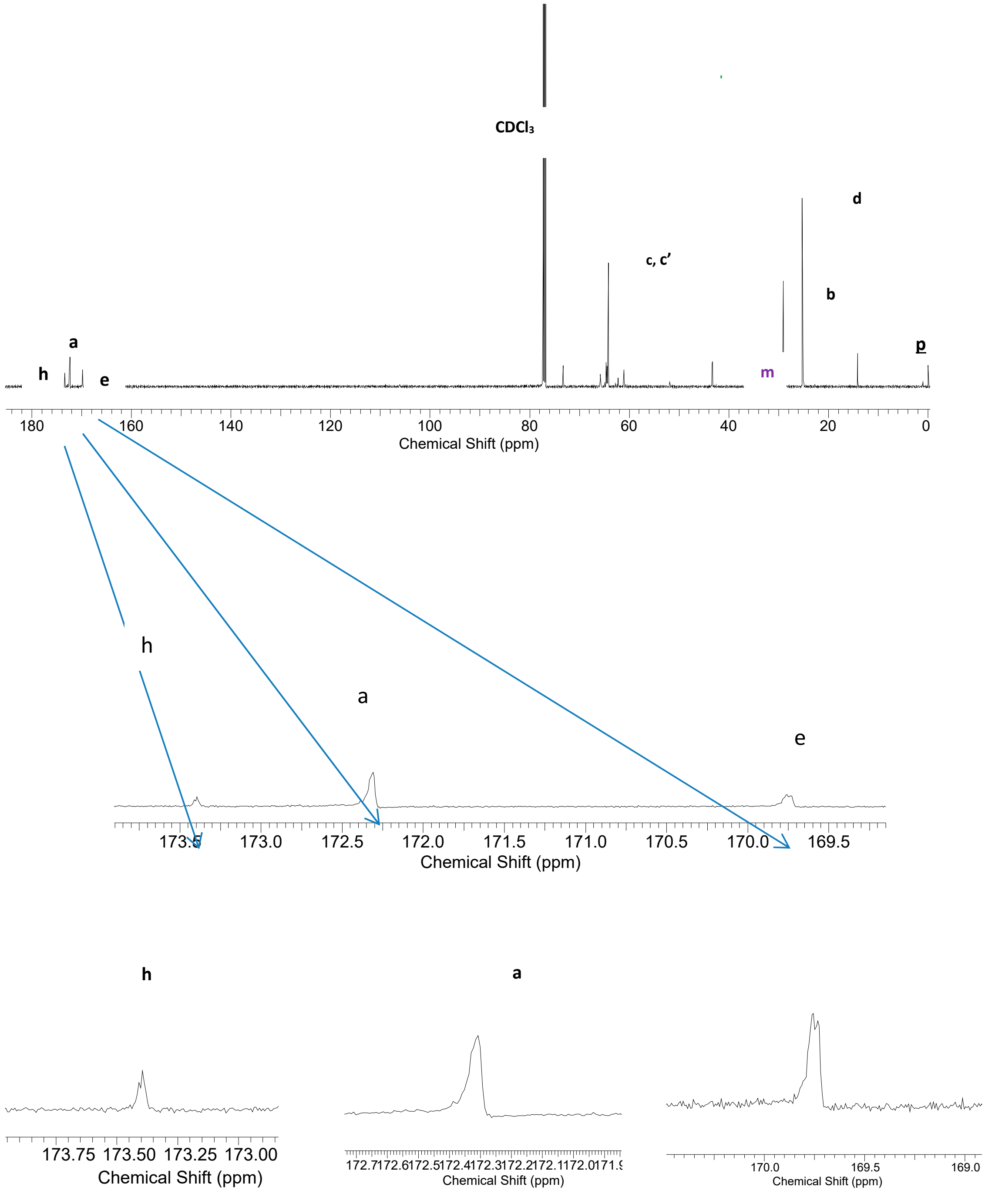

Figure S2. ^{13}C NMR spectra of poly(butylene succinate-co-butylene citrate) – polymer NG48. Carbonyls carbon signals of; e, h – butylene citrate units, a – butylene succinate

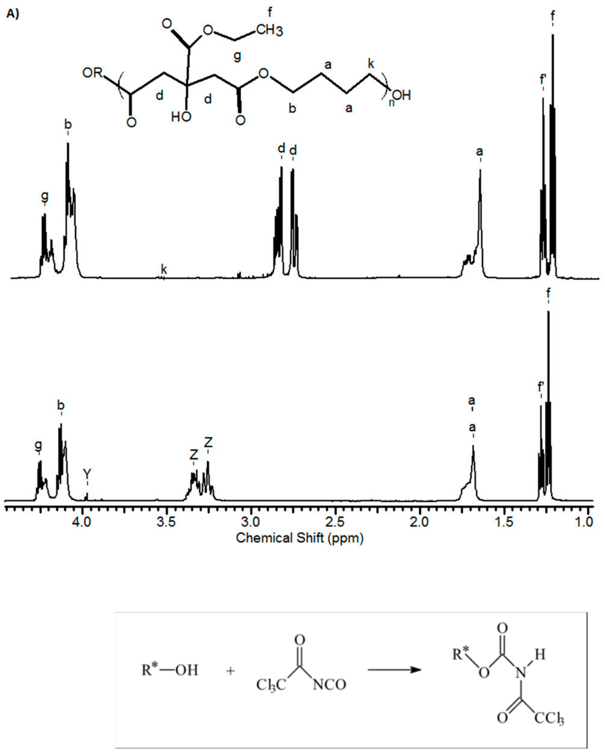

Figure S3. ^{1}H spectra of poly(butylene citrate) before reaction with trichloroacetyl isocyanate, an after reaction

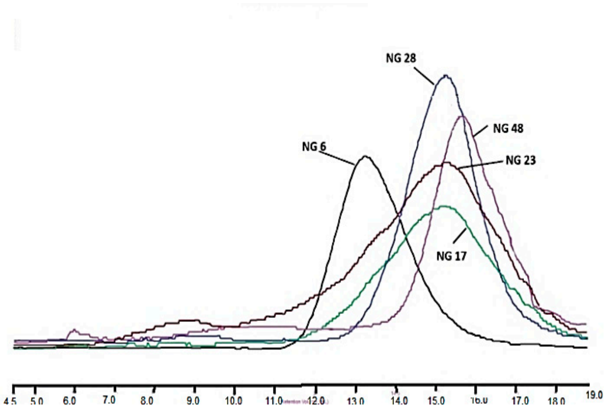

Figure S4. GPC elugrams of the synthesized butylene succinate / butylene citrate copolymers

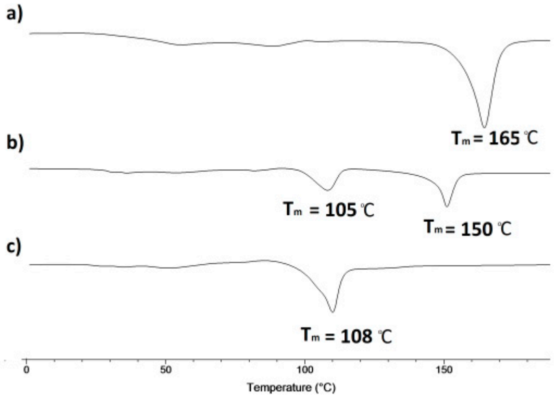

Figure S5. DSC thermograms of poly(L-lactide)-block-poly(butylene succinate) - I run a) NG7, b) NG8, c) NG 9 copolymers

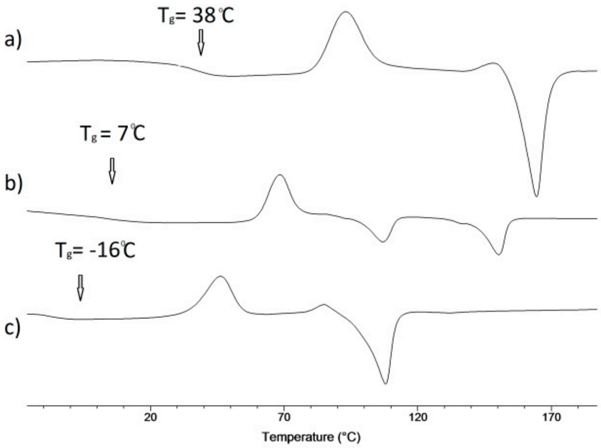

Figure S6. DSC thermograms of poly(L-lactide)-block-poly(butylene succinate) - II run a) NG7, b) NG8, c) NG 9 copolymers

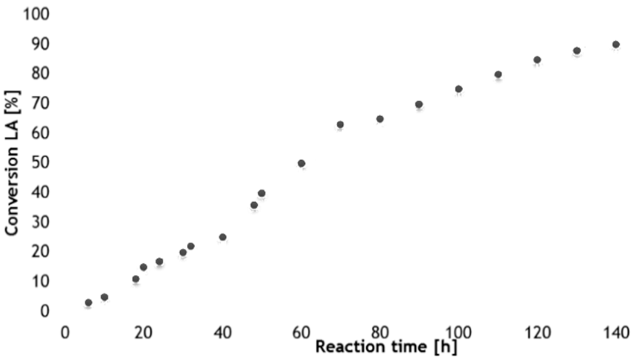

Figure S7. Dependence of L-lactide conversion and reaction time during L-lactide polymerization conducted in the presence of poly(butylene citrate) – NG48 initiator

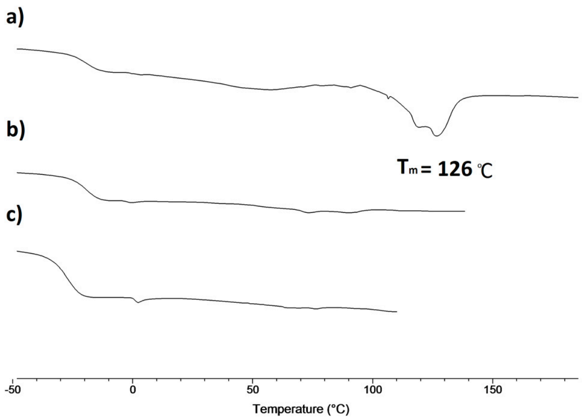

Figure S8. DSC thermograms of poly(l-lactide)-block-poly(butylene succinate-co-butylene citrate) –1st run, sample; a) NG 51, b) NG 53, c) NG 55

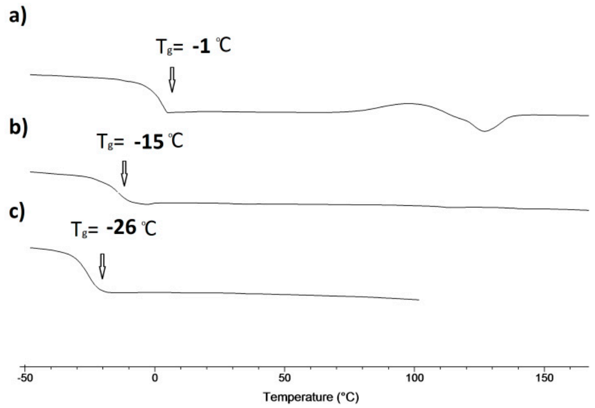

Figure S9. DSC thermograms of poly(l-lactide)-block-poly(butylene succinate-co-butylene citrate) – 2nd run, sample; a) NG 51, b) NG 53, c) NG 55

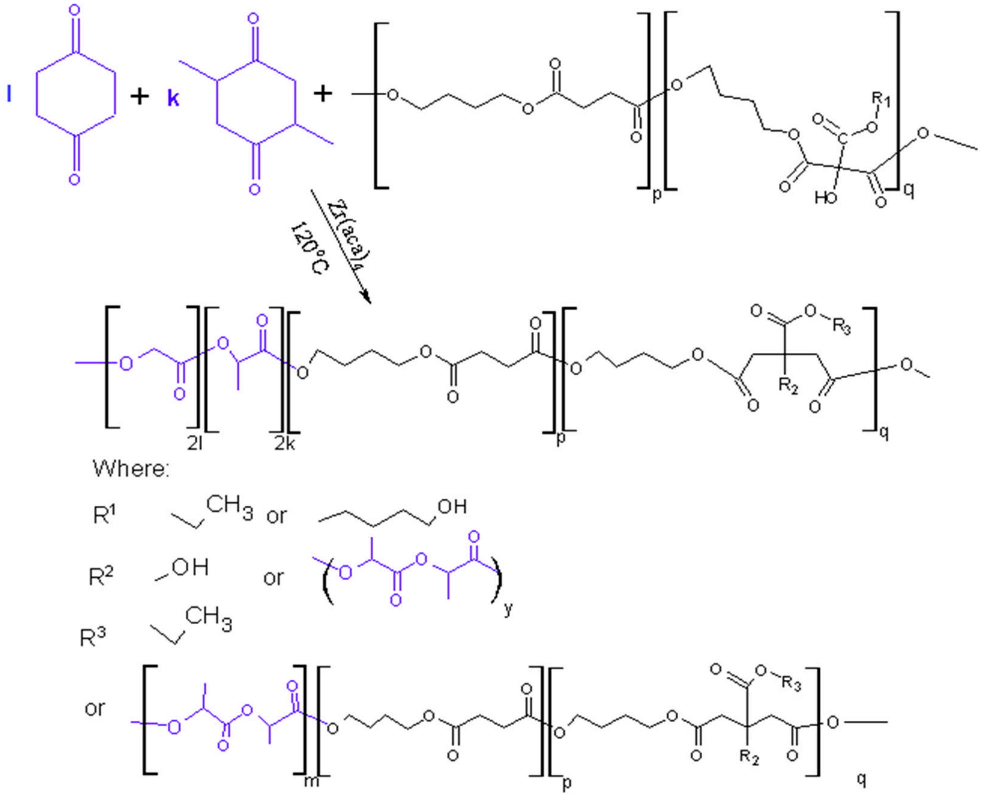

Scheme S1. Course of copolymerization of L-lactide with glycolide using poly(butylene succinate-co-butylene citrate) as reaction macroiniator
